# The Role of Language in Structuring Social Networks Following Market Integration in a Yucatec Maya Population

**DOI:** 10.3389/fpsyg.2021.656963

**Published:** 2021-12-16

**Authors:** Cecilia Padilla-Iglesias, Karen L. Kramer

**Affiliations:** ^1^Department of Anthropology, University of Zurich, Zurich, Switzerland; ^2^Leverhulme Centre for Human Evolutionary Studies, Department of Archaeology, University of Cambridge, Cambridge, United Kingdom; ^3^Anthropology Department, University of Utah, Salt Lake City, UT, United States

**Keywords:** bilingualism, Maya, market integration, behavioral ecology, cultural evolution, social networks, language shift, Mexico

## Abstract

Language is the human universal mode of communication, and is dynamic and constantly in flux accommodating user needs as individuals interface with a changing world. However, we know surprisingly little about how language responds to market integration, a pressing force affecting indigenous communities worldwide today. While models of culture change often emphasize the replacement of one language, trait, or phenomenon with another following socioeconomic transitions, we present a more nuanced framework. We use demographic, economic, linguistic, and social network data from a rural Maya community that spans a 27-year period and the transition to market integration. By adopting this multivariate approach for the acquisition and use of languages, we find that while the number of bilingual speakers has significantly increased over time, bilingualism appears stable rather than transitionary. We provide evidence that when indigenous and majority languages provide complementary social and economic payoffs, both can be maintained. Our results predict the circumstances under which indigenous language use may be sustained or at risk. More broadly, the results point to the evolutionary dynamics that shaped the current distribution of the world’s linguistic diversity.

## Introduction

Market integration in indigenous communities often leads to the adoption of norms and practices, including language, from the cultural majority (Kandler et al., 2010; Isern and Fort, 2014). This adoption is commonly regarded as symptomatic of an acculturation process where previous means of subsistence are gradually replaced with market activities and is accompanied by inevitable cultural and linguistic erosion (Fishman, 1991; Makihara, 2004; Garrett, 2005; Godoy et al., 2007; Hamel, 2008; Veile et al., 2014; Mattison and Sear, 2016; de León, 2019). However, few studies have quantified how or why such erosion occurs. Consequently, a comprehensive account is lacking of the social and economic factors that drive individual decision-making about language learning, use, transmission, and how these factors change following market integration. Yet, these dynamics are vital to predict the long-term fate of linguistic environments, and more broadly to understand the vast diversity that characterizes language, one of the defining traits of our species.

Humans are socially organized into groups whose members share norms, expectations, skills, and beliefs through common codes of communication (Foley and Mirazón Lahr, 2011). Several researchers have proposed that language differences are among the most salient indicators of group identity and behaviors (Nettle, 1997; Dunbar, 2003; Cohen, 2012; Moya, 2013). Several reasons have been suggested why people assort with others from the same linguistic group. Among them, a common means of communication is indispensable for successful coordination between individuals, and following on this, linguistic boundaries are thought to be key in preventing the diffusion of institutions between groups and instrumental in maintaining cultural diversity (Richerson et al., 2016). Assorting with the same-language speakers further allows individuals to minimize coordination costs by interacting with others who share the same social norms, preferences, or expectations (McElreath et al., 2003).

Language also shape many social behaviors. Extensive historic and ethnographic evidence supports that linguistic boundaries delineate reciprocity, marriage, and risk-sharing networks (Wiessner, 1983; Nettle, 1998, 1999) and motivate social distancing, stereotyping, and political action (Schieffelin et al., 1998; Irvine et al., 2000). Laboratory experiments show that United States infants prefer to befriend, learn from, or exchange toys with native-language speakers rather than foreign-language speakers (Kinzler et al., 2009, 2011; Kinzler and Dautel, 2012). However, the universality of language boundaries in structuring social behaviors and cooperative or collaborative partnerships has become increasingly disputed because many ethnic groups are linguistically heterogeneous or linguistic boundaries are not socially meaningful (Hill, 1978; Foley, 1986; Aikhenvald, 2003; François, 2012). Investigating what shapes an individual’s linguistic repertoire in determining social behaviors is essential to understand the consequences of acquiring competencies in one or more languages, particularly in the context of changing socioeconomic landscapes.

Similarly, although indigenous languages are often assumed to be replaced by majority languages during market integration, a few studies show that this process is nuanced. For example, among the Arctic Sámi, a recent transition from hunting and gathering to a periodic engagement in tourism has led to a “seasonal multilingualism” (Pietikäinen, 2015, 2018). Since the Arctic became a popular tourist destination, Russian, German, English, Japanese, and French are now used by the Sími during some times of the year (Pietikäinen, 2010). Although the outcome is multilingual language use, Sámi languages are far from disappearing. Rather, they have experienced an unprecedented revitalization as they become linked to a source of income and pride and are an index of ethnic difference and authenticity.

In *mixed economies* (those that combine long-standing subsistence practices with some wage labor or other market enterprises), languages associated with local and non-local social networks may serve different, yet complementary functions. Local networks often are a crucial social asset in providing access to labor pools and buffering against resource shortages caused by failures in food production or illness. Over the course of market integration, there is often a shift to reliance on formal institutions, such as banking and credit, and on non-kin for support. However, these forms of assistance are often not a reliable strategy to offset resource shortfalls, and within-group relationships remain important in populations integrating to national markets (Pisor and Gurven, 2016; Hackman and Kramer, 2021a). Similarly, market exchange with out-groups may expand the cushioning effect that local networks provide, such that non-local resource access can buffer against shocks that may impact all local network members (Baggio et al., 2016; Pisor and Gurven, 2017). While some anthropological studies have quantified the effects of mixed economic strategies in maintaining indigenous cultural norms (Bunce and McElreath, 2017, 2018), such investigations have not included language (see Grin, 1995; Grin and Vaillancourt, 1997).

We emphasize three key points about market integration and language. First, market integration can be viewed as a new arena where interactions between groups accelerate. Second, a shared language is indispensable for successful coordination between individuals and to identify cultural affiliation. If individuals from different language groups interact, we propose they may consider both language functions (coordination and identity) when making decisions about what languages to learn, use, and transmit in particular contexts. Third, to predict whether the indigenous language will be replaced by the majority language, or the two will be used in tandem, we need to investigate the factors determining when, how, and how much individuals interact with members of the majority culture and the terms under which such interactions take place.

In this paper, we ask how new subsistence opportunities affect local social organization in a rural Yucatec Maya community, and in turn, how language use responds. Over a 27-year period and data spanning the transition from a subsistence to a market-integrated community, we first investigate how changing livelihoods are associated with a change in the use of indigenous and majority languages, and whether economic returns vary across individuals. Second, using in-depth social network analyses, we evaluate the social outcomes of speakers with different linguistic repertoires. Models of the possible outcomes that economic change has for linguistic diversity are abundant as is research on the potential for language to guide social behaviors. However, this is the first attempt we are aware of that integrates both social and economic payoff matrices into a single analytical framework to gain a more informed picture of factors guiding individual language use. Such integration is necessary to better understand the historic dynamics that shaped the current distribution of the world’s tongues and to predict the sustainability of linguistic diversity following ongoing globalization and market integration that affect many small-scale indigenous populations today.

## Materials and Methods

### Maya Study Site

The Maya study population is located in a remote area of the Puuc region in the interior of the Yucatan Peninsula, Campeche, Mexico. The indigenous Yucatec Maya who inhabit this rural area live in small villages of subsistence maize farmers and in a few market and administrative towns. Longitudinal economic, demographic, and social data have been collected in one community by the second author since the early 1990s (Kramer and Boone, 2002; Lee and Kramer, 2002; Kramer, 2005; Kramer and Veile, 2018). Prior to 2000, all community members were subsistence maize farmers, the household was the unit of production, and each family grew its own food. Because transportation to market towns was limited, there was little means to engage in the regional economy, surplus crop production, schooling, or wage labor. The distribution of land across households was relatively homogenous due to the lack of market opportunities and to an institutionalized land tenure system (*ejido*) where the collective ownership of agricultural lands guarantees all community members relatively equal and adequate access to food, firewood, and other resources. The study community’s 15,833-hectare *ejido* allotted following the Mexican Revolution land reforms, has been sufficient to support this small but growing population (the study population currently consists of ∼550 individuals) for the past 65 years.

In the early 2000s, economic development began rapidly when a paved road was built, which facilitated access to new farming methods, the transportation of crops to the market, children to schools, and people to wage labor jobs. These changes have expanded the ways in which households make their living, the amount of schooling children receive, exposure to Spanish speakers, and increased social interactions with non-Maya and individuals of different political, social, and wealth statuses.

The majority (∼80%) of the Maya diet still comes from maize produced by the household. The region where the Yucatec Maya live is characterized by low population density and a high degree of ethnic homogeneity. Marriages between Maya and non-Maya are not common, nor is immigration into the region (INEGI, 2019).

In contrast to what many have regarded as inevitable acculturation (Mijangos-Noh, 2001; Gurven et al., 2015; Mattison and Sear, 2016), social and residential structures in many rural Yucatec Maya communities have remained strikingly stable (Gaskins, 2003; Kramer, 2005), as they have in this one. To supplement agriculture returns, most families engage in part-time, seasonal or occasional wage labor to generate cash for school supplies and fees, market goods, medical costs, or in times of need. However, very few families have abandoned farming altogether. The majority of wage labor is unskilled farm, construction or maquiladora work in towns and enterprises within a few hours of the community. In these cases, Maya is spoken among laborers, while Spanish is spoken with employers. If a household has several sons over the age of 15, usually one works in part-time wage labor, while others maintain the family’s agricultural base. By 2017, in 30% of households, 50% of adult family members (age > 15) were engaged in some forms of part-time wage labor (Kramer et al., 2021). In 7% (*n* = 6) of the households, the head of a household worked in full-time wage labor (most as shop keepers or agricultural engineers), where proficient Spanish is needed.

Mayan is a child’s first language, and the language spoken at home, and used for work, play, and social interactions among both adult and child community members. Spanish is rarely heard within the community, except in the classroom. Rural Campeche is ethnically quite homogenous, and outside the community, Mayan is also commonly spoken among ethnic Maya in neighboring market towns. In larger towns and with government officials, Spanish is spoken. Consequently, there is individual variation in Spanish use depending on time spent outside the community, and in what capacity–as wage laborers, or government officials or committee representatives. Men, for example, spend more time outside the village than women, a trend that has changed little over the study period (Schacht et al., 2018).

Within the community, local schools have been built for grades 1–9 in the last decade. Most children now attend at least grades 1–6 and learn to read and write. About 50% of teens elect to attend high schools in other towns where they board and return home on the weekend. Children are initially exposed to Spanish, the official Mexican national language, when they start to attend the local primary school. Although schools are now administered under the “Intercultural Bilingual Education” legislation, which stipulates that 30% of the class time be taught in Maya and 70% in Spanish (Santibañez, 2016), in reality, as reported for other villages in the region (de León, 2017; Osorio-Vázquez, 2017), there are some deviations from this proscription. The youngest children are instructed in Maya, with the amount of Spanish spoken in the classroom increasing with grade. Most textbooks are written in Spanish, accompanied by some local histories written in Mayan. While not all teachers are Mayan speakers, Mayan is principally employed for classroom management while Spanish is the language of instruction. During recess and play, children speak among themselves in Maya.

### Hypothesis and Predictions

Recently, economic development has resulted in an increased connectivity between previously isolated rural Yucatec Maya villages and larger market towns. This has led to greater opportunities to supplement traditional subsistence agriculture with market-based activities, where Spanish may be predominantly used. Because households are the unit of production and consumption, they may benefit from having at least *some* Spanish-speaking members who can engage in market opportunities while other members maintain the agricultural base (what we refer to as mixed economy; Hackman and Kramer, 2021b). Similarly, Mayan remains indispensable for *all* individuals to coordinate with local community members and to signal ethnic affiliation to participate in and benefit from participation in local exchange and redistribution networks.

We first describe long-term language trends following socioeconomic changes related to an increased exposure to market opportunities and Spanish. Then, drawing from our hypothesized relationships between the market integration and language use, we make the following predictions:

*P1:* Given the market benefits of the majority language, we expect an increased trend toward bilingualism over the course of market integration. However, we do not expect the majority language to replace the indigenous one, given that each language provides individuals and households with access to different yet valuable social and economic spheres.

*P2*: Individuals who live in households with less arable land, or in larger households are expected to diversify their economic strategy such that some members engage in wage labor. If indeed competence in the majority language allows them to engage in wage labor (and hence their households to adopt a mixed economic strategy), they should be more likely to be Spanish speakers.

*P3*: If both the majority and indigenous language serve complementary, yet, valuable functions we do not expect to observe language-based homophily (or preferential association) among Spanish speakers in community social networks.

*P4*: We expect that engaging in a mixed economy is both more socially desirable and economically profitable for households than exclusively relying on subsistence agriculture or completely shifting to wage labor (Hackman and Kramer, 2021a). In addition, and tied to P3, if languages serve complementary functions, we expect that having members with competences in both languages is advantageous and/or that such advantages depend on an individual’s the personal attributes.

### Determining Linguistic and Economic Changes Across the Process of Market Integration

To determine whether language use changes over the course of market integration and its association with economic changes, we used the data collected at two time points 27 years apart. Baseline linguistic and socioeconomic data were collected in 1992 (*n* = 293 individuals and *n* = 57 households), the first year of study in this community and prior to the introduction of a paved road and market integration. Follow-up data were collected in 2017 (*n* = 545 individuals and *n* = 142 households). The same methods were employed at both time points; data were collected by the second author (see Supplementary Table 2 for descriptive statistics at each time point). Age and sex were also recorded in annual censuses that have been collected since 1992.

The individual characteristics included here include language use and education for all individuals over the age of 6, as well as the wage labor status for those over the age of 16. Language proficiency was determined through observation and by interviewing household members. *Spanish fluency* was coded as a binary variable, where 1 indicates being fluent and 0 indicates no or very little Spanish competency. A binary term was employed for two reasons. First, because we wanted to assess both predictors of speaking Spanish within time periods, and whether speaking Spanish predicted certain social and economic outcomes, a binary outcome is statistically most appropriate for generalized linear mixed models (GLMMs) (see sections Analyses of the “Structure of Social Networks,” “Analyses of Social Network Outcomes,” and “Results”). Second, a significant strength of our study is the ability to capture sociolinguistic change over generational time (such as language shift), which means the data needed to be expressed compatibly. In 2017 language competence was collected on a four-point continuous scale (where 0 corresponds to “no competence at all,” 1 corresponds to “very little,” and 2–3 corresponds to “conversational” and “fluent,” respectively). In 1992 the data were collected as a binary variable (where 0 corresponds to either none or little and 1 corresponds to “conversational” or “fluent”). By binarizing the 2017 data, and assigning “1” to those who were at least conversationally fluent in Spanish, we could maximize the comparability of our data and analyses across cohorts, and assess changes in the number of competent Yucatec Mayan and Spanish speakers in each time period. *Educational level* was recorded as the number of years of completed education (from primary school onward) by an individual. *Wage labor status* was coded as a binary variable, where 1 indicates that the individual engaged in wage labor (regardless of whether they also engaged in agricultural work) and 0 indicates that the individual did not engage (Supplementary Table 1).

We also recorded each individual’s *household characteristics*, including economic strategy, agricultural wealth, and household size. Household *economic strategy* was coded as a categorical variable, where 1 indicates a *mixed strategy* where the male household head (MHH) engaged in part-time wage labor in addition to agricultural work, 2 indicates a committed wage labor strategy where the MHH exclusively engaged in wage labor, and 0 indicates a committed agricultural strategy where the MHH was engaged exclusively in agricultural activities (no female household head (FHH) was a wage laborer at either time points). *Agricultural wealth* is measured as the number of hectares a household had under cultivation.

In 2017, we have precise information on the income that households obtained from the different economic activities in which their members participated. Hence, we recorded the *agricultural income, wage labor income* obtained by each household. We also calculated the *net income* of each household, and included this in our analyses as a measure of overall wealth. The net income of a household was calculated as the sum of all the cash income a household received (income from agriculture, wage labor, and government aid), minus the expenses of the household (agricultural expenses as well as any other living expenses). *Household size* was defined as the number of children ever had by the FHH.

### Social Network Data

To evaluate the social network outcomes of speakers with different linguistic repertoires as well as whether same-language speakers are preferentially assorted with one another, ego-centered social networks were drawn for all FHHs (*n* = 86) in 2017. The analytic focus is on household-level outcomes according to the linguistic repertoire of their members. Because women spend the majority of the time in the village (Cashdan et al., 2016) and are generally responsible for household duties including childcare, we limit our analyses here to household-level networks reported by female egos.

Three types of networks were used in the analyses: (1) who in the village did the FHH visit weekly; (2) who in the village helped the FHH with childcare and food^1^; and (3) who in the village would egos be willing to lend a sum of money equivalent to a week’s salary.

Networks 1–3 are referred to as “visiting,” “helping,” and “money” networks, respectively. The visiting network corresponds to a non-costly associative network, which may simply reflect the residential structure of the village, meaning that those living proximate to each other may be more likely to visit each other’s households. This network is important as a baseline to make sure that the observed patterns in the other networks are not the result of the spatial availability of potential interaction partners. People may seek in-group members in many types of activities, but particularly in helping interactions when defections and coordination failures are costly, and the benefits to others are large (Moya and Boyd, 2015). The helping network represents a costly local cooperative network, while the money network is a novel network arising as a consequence of market integration and access to cash.

Female household heads (egos) could nominate an unlimited number of alters. Ties were directed, which allows ego to declare cooperating with an alter but not receiving cooperation in return. For the money network, because cash income is distributed within the household and husbands and wives have an open relationship with respect to money, the question was asked to both female and male household heads. Because the aim is to capture between household ties, if a FHH nominated an alter from their household, these ties were removed from the analysis. This means that all MHHs and FHHs could be potential alters for all households except their own. For social network analyses, individuals younger than age 12 years were excluded as they generally do not participate much in community life beyond their households or make significant economic contributions (see Supplementary Table 2 for descriptive statistics).

### Analyses of the Structure of Social Networks

To assess whether there was positive assortment (homophily) based on language (P3), assortativity coefficients were calculated for each of the networks (Newman, 2010). Permutation tests generated a null distribution of assortativity coefficients by simulating 1,000 random networks with the same properties as the real networks: that is, the same numbers of help, visits, or money partners, the same number of connections to egos, and the same proportion of people competent in each of the languages. In addition, Jaccard indices were computed to quantify the similarity between the visit, help, and money networks (Jaccard, 1908). A Jaccard index *J*(*A*, *B*) [where 0 ≤ *J*(*A*, *B*) ≤ 1] measures the proportion of links shared between the binary networks *A* and *B*, compared to the links in either network. The indices closer to 1 indicate greater similarity.

**FIGURE 1:**
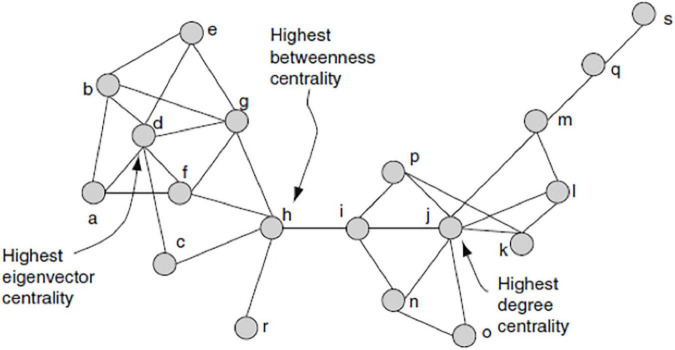
Adapted from https://ultrabpm.wordpress.com, the household-level social network measures used in the present analyses is shown. “h” represents the node with the highest betweenness centrality, “d” that with the highest eigenvector centrality (EC), and “j” that with the highest degree centrality. Unlike the networks in our study, the connections shown in the figure are not directed, meaning that there is no distinction between in- and out- degree.

**FIGURE 2:**
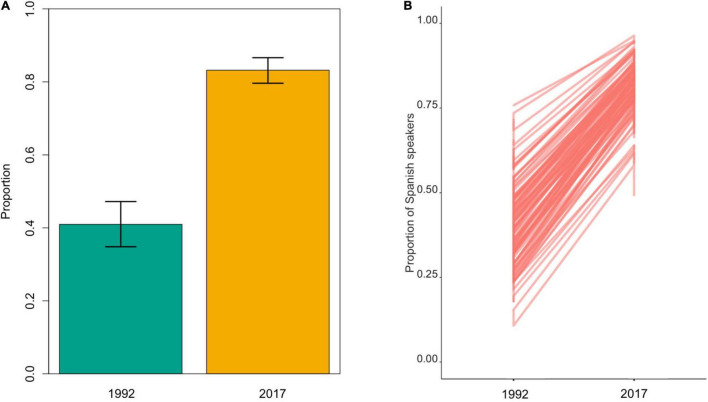
On the left **(A)**, posterior means (bars) and 90% highest posterior density intervals (HPDIs; error bars) from the Bayesian generalized linear mixed models (GLMMs) predict the average proportion of Spanish speakers in the village setting sigma household to 0. Setting the SDs for the varying intercepts (sigma household) to 0 produces predictions for an average household but ignores uncertainty arising from variation among households. On the right **(B)**, the results from the simulation of 100 households with unique intercepts sampled from the posterior distribution. This produces predictions that account for a variation among households.

To evaluate whether the linguistic repertoire of household members affected the network position of their households, we calculated three measures (Figure 1). (A) Network size (in- and out-degree), which refers to the total number of incoming and outgoing ties a household had (Freeman, 1978). (B) Betweenness centrality, which is proportional to the number of geodesic (shortest) paths a node lies on between any other two nodes (Newman, 2010). Higher betweenness scores indicate that a household acts as a bridge or broker between otherwise unconnected households (Freeman, 1977). (C) Eigenvector centrality (EC) takes into account both the number and degree centrality of the ties of a node. Nodes connected to other well-connected nodes have a higher EC values, as do nodes with many neighbors. Households with high EC had a large number of partners, who themselves had a large number of partners (Wasserman and Faust, 1994; Figure 2).

### Analyses of Social Network Outcomes

Table 1 summarizes the empirical questions addressed by the predictions, and their outcome variables. For all statistical analyses, we used Bayesian inference. In a Bayesian framework, each model conditions its data on prior probability distributions and uses Monte Carlo sampling methods to generate posterior distributions for its parameters. This permits comparisons between posterior distributions across sexes, age groups, occupational, or linguistic categories without requiring specific *post hoc* tests, and it obviates the need to adjust for multiple comparisons (Gelman et al., 2012). Bayesian inference also allows for better interpretation of differences between parameter estimates relative to a specific value by obtaining the entire posterior distribution for each predictor. It also gives the highest posterior density intervals (HPDIs), which reveal the narrowest interval containing the specified probability mass. Regularizing priors, which are more conservative than the implied flat priors of non-Bayesian procedures, were adopted to prevent the model from overfitting the data (McElreath, 2015). Having fit alternative parameterizations for all models, we believe that the results are qualitatively robust to changes in priors.

**TABLE 1:** Summary of the empirical questions addressed with each prediction and the outcome variable used to test it.

Prediction	Questions addressed	Outcome variable
P1	(a) Has the proportion of Spanish-speakers in the village increase from 1992 to 2017 at the expense of Yucatec Mayan speakers? (b) Has the proportion of Spanish speakers per household changed from 1992 to 2017?	(a) Number of Spanish speakers out of all individuals over 6 years of age in the village. (b) Number of Mayan speakers out of all individuals over 12 years of age in the village* (c) Number of Spanish speakers per household out of all individuals over 6 years of age in the household
P2	(a) Are individuals from larger families or families with little land more likely to speak Spanish? (b) Does speaking Spanish predict whether an individual will adopt a wage labor strategy?	(a) Probability of an individual over 12 years of age of speaking Spanish in 1992 and 2017 (b) Probability of an individual over 16 years of age of engaging in wage labor in 1992 and 2017
P3	Do Spanish-speaking households assort with one another?	Assortativity coefficient on Spanish in visiting, helping and money networks
P4	(a) Does having Spanish-speaking members affect households’ social network position? (b) Is the effect of Spanish-speakers on households’ social network position network-dependent or dependent on speakers’ personal attributes?	(a) Households’ in-degree centrality in visiting, helping and money networks in 2017 (b) Households’ eigenvector centrality in visiting, helping and money networks in 2017 (c) Households’ betweenness centrality in visiting, helping and money networks in 2017

*Changes in the number of Mayan speakers across cohorts as well as predictors of competences in Yucatec Mayan were not analysed statistically as all villagers were competent in Yucatec Mayan, and therefore there was no variability.

Our analysis proceeds in several steps. First, to assess whether over the course of market integration between 1992 and 2017, Yucatec Mayan has been abandoned in favor of Spanish (P1), Bayesian GLMMs with a logistic link function (Hosmer et al., 2013) were used to predict (P1a) if the proportion of villagers who spoke Spanish had changed and (P1b) if the proportion of Spanish speakers per household had changed. We did not statistically analyze whether the proportion of Mayan speakers in the village had changed as because all villagers were competent in Yucatec Mayan at both time periods. Second, to evaluate whether linguistic decisions were tied to market integration (P2), we examined the relationship between language and socioeconomic variables that measure the level of market integration. This approach allows us to further test the direction of causality; in other words, we test whether new job opportunities emerging from market integration were associated with decisions about which language(s) to learn and transmit across time. Bayesian GLMMs with a logistic link function are used to predict whether within-time periods, the probability that an individual speaks Spanish was affected by the household wage labor strategy, after controlling for the educational attainment.

We also used Bayesian GLMMs with a logistic link function to examine which factors affect the probability of an individual engaging in wage labor (P2b). For this set of models, only individuals of age 16 and older were included (younger teens do not engage in wage labor). For all analyses, except those assessing the proportion of Spanish speakers per household, random intercepts for “household” were included to account for the data’s nested structure and associated clustering.

Thired, Bayesian inference was used for the analysis of social networks (P4). To predict in- and out-degree as a function of household characteristics (see section “Social Network Data”), in this case we used Bayesian Generalized Linear Models (GLMs) with a Poisson link function due to the count nature of the response variables. In models predicting households’ EC or BC, a Gaussian link function was used. Random intercepts for “household” were not included since these analyses are performed at the household level (each data point represents a single household).

Forth, testing P4 required evaluating not only the social but also the economic returns for adopting a mixed economic strategy. Here we assessed whether households engaged in wage labor had a higher net income than those who did not. To assess this, we fit Bayesian models with a Gaussian link function to predict a household’s net income as a function of the economic strategies adopted by its members. We fit two models, one including the Spanish competence of the FHH and MHH and another without these competences.

For all models, continuous parameters were *z*-transformed (their means were subtracted and divided by their SDs within each time point) to allow for coefficient comparability. Before conducting our analyses, we checked for multicollinearity among predictors using the generalized variance inflation factor (GVIF). All GVIF values fell below the lowest commonly recommended threshold of 2, indicating that our models should not suffer from multicollinearity (Zuur et al., 2010).

The parameter estimation was achieved with RStan (Stan Development Team, 2016), running three Hamiltonian Monte Carlo chains in parallel until convergence was suggested by a high effective number of samples and *R*^ estimates of 1.00 (McElreath, 2015). In some cases, this entailed 5,000 samples per chain and in others 10,000. In the former case, we used 1,000 as warm-up and in the latter 2,000. We also visually inspected trace plots of the chains to ensure that they converged to the same target distributions and compared the posterior predictions to the raw data to ensure that the models corresponded to descriptive summaries of the samples.

For model comparisons, we used the Widely Applicable Information Criteria (WAIC), which provides an approximation of the out-of-sample deviance that converges to the leave-one-out cross-validation approximation in a larger sample (Gelman et al., 2013). We also calculated model weights—the probability that a given model will perform the best on new data, relative to other candidate models (McElreath, 2015). Analyses were performed in R 3.5.2 using the *brms* package (Bürkner, 2017; R Core Team, 2018). We present a complete description and justification of the priors, model specifications, model comparisons, and model coefficients in S1 Text. In addition, *igraph* and *proxy* packages were utilized for the manipulation and analysis of the networks (Csardi and Nepusz, 2006; Meyer et al., 2019).

## Results

### Has the Linguistic Landscape Changed Following Market Integration?

The proportion of fluent Spanish speakers in the village has doubled from 1992 [mean = 0.41, 90% HPDI: (0.36, 0.46)] to 2017 [mean = 0.83, 90% HPDI: (0.81, 0.86)] (Figure 2). However, this increase was not at the expense of Yucatec Mayan. There were no monolingual Spanish speakers in either periods, i.e., all Spanish speakers being bilingual.

The proportion of Spanish speakers per household increased evenly across the households in the village [1992: mean = 0.50, 90% HPDI: (0.46, 0.54); 2017: mean = 0.83, 90% HPDI: (0.81, 0.86)], which indicates that the trend has been homogeneous across the households. In 1992, 82% of the households (*n* = 32) had at least one fluent Spanish speaker, while in 2017 this increased to 98% (*n* = 54).

### Is Spanish Speaking Associated With Household Economic Strategy?

In 1992, 1.87% of people over the age of 12 (*n* = 4) lived in households committed to wage labor strategy, and 11.26% (*n* = 24) in the households pursuing a mixed economic strategy. In contrast, 5.78% (*n* = 30) and 39.50% (*n* = 205) in 2017 lived in households engaged in committed wage labor and mixed economic strategies, respectively. To test whether there was a direct association between household-level economic strategy and linguistic behavior, we inspected sources of variation within each time period.

Of the six Bayesian GLMMs fitted to predict whether an individual over 12 is fluent in Spanish in 2017 (and not in 1992), the top-performing model included an interaction between an individual’s age and the size of the individual household (Supplementary Table 4). This means that besides an inverse relationship between an individual’s age and probability of speaking Spanish [log-odds=−0.94, 90% HPDI: (−1.27,−0.57)], younger individuals had an even greater probability of speaking Spanish when they came from large families [log-odds =−0.61, 90% HPDI: (−1.09,−0.14)] (Figure 4).

In 2017, living in a household where the MHH is a committed wage laborer significantly increased an individual’s chances of being bilingual [log-odds = 1.62, 90% HPDI: (0.28, 2.95)]. In contrast, agricultural *wealth* did not influence an individual’s probability of speaking Spanish [log-odds = 0.29, 90% HPDI: (−0.06, 0.66)].

**FIGURE 3:**
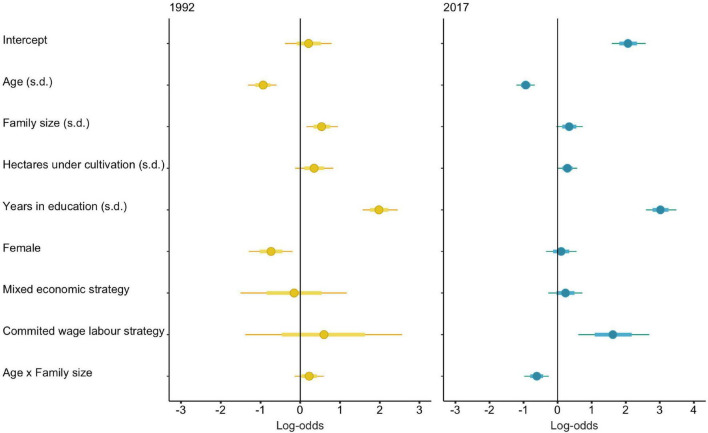
Log-odds from the posterior distribution of the full multilevel logistic mixed model showing the probability that an individual spoke Spanish in 1992 **(left)** and 2017 **(right)**. Points show median probability that an individual spoke Spanish, thick lines are 50% HPDIs, and thin lines are 90% HPDIs. A vertical line represents no effect. All continuous parameters are standardized within each time period. The effects of a mixed economic strategy and of a committed wage labor strategy are reported in relation to the effects of a committed agricultural strategy (the reference factor).

The disappearance of the male-bias in bilingualism is likely mediated by educational attainment, which was a strong predictor of bilingual competence [Figure 3; log-odds = 3.02; 90% HPDI: (2.45, 3.59)] and had disproportionately increased in men. The mean number of completed years of education in 1992 for individuals aged 12 and older was 3.80 for men (SD = 2.40) and 3.14 for women (SD = 1.91). In 2017, it was 8.34 for men (SD = 3.70) and 6.50 for women (SD = 3.64). While there is no evidence for sex differences in schooling in 1992, this is no longer the case in 2017. When running the same model for 2017 without including “Years in education” as a predictor, being female significantly decreases the probability of speaking Spanish [log-odds =−0.46, 90% HPDI: (−0.89,−0.04)], while the rest of the coefficients do not significantly change. By 2017, 74.4% (*n* = 160) of men were fluent in Spanish compared to 63.7% (*n* = 130) of women.

**FIGURE 4:**
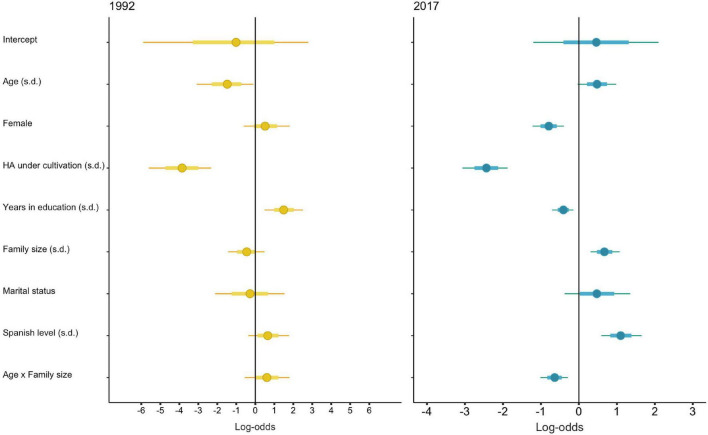
Log-odds from the posterior distribution of the full multilevel logistic mixed model showing the probability that individuals over 16 years of age engaged in wage labor in 1992 **(left)**. and 2017 **(right)**. Points show median probability that an individual engaged in wage labor, thick lines are 50% HPDIs, and thin lines are 90% HPDIs. A vertical line represents no effect. All continuous parameters are standardized within each time period.

In 2017 (but not in 1992), the best model for predicting the probability that an individual would engage in wage labor includes the interaction between an individual’s age and household size (Supplementary Table 5). This result shows that young adults were more likely to engage in wage labor only when coming from large families [log-odds =−0.64, 90% HPDI: (−1.10,−0.16)] (Figure 4).

Moreover, in 2017, adults living in families with less land were more likely to work for wages [log-odds =−0.41, 90% HPDI: (−0.77,−0.04)] and in both 1992 and 2017, women were less likely to engage in wage labor than men [log-odds =–3.87, 90% HPDI: (–6.05,–1.85) and log-odds =–2.44, 90% HPDI: (–3.21,–1.67), respectively] (Figure 4). The lack of significant predictors of working for wages in 1992 reflects the overall rarity of doing so (8 out of 134 individuals considered did so), and thus most likely is the result of limited statistical power.

Then, we examined the wage labor status of all individuals over the age of 12 in 2017 (Supplementary Table 3) and found that *none* of the Mayan monolinguals were exclusively wage laborers. Only 3% (*n* = 2) of them engaged in any kind of wage labor when compared to 22.8% (*n* = 68) of bilinguals, further highlighting the relationship between language and subsistence.

### Do Spanish Speaking Households Assort With One Another?

**FIGURE 5:**
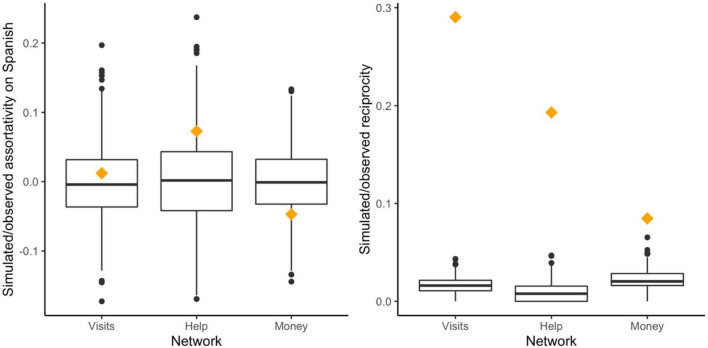
On the **(left)**, assortativity on whether individuals speak Spanish or not for the three social networks is shown. On the **(right)**, reciprocity for the three social networks is shown. Orange diamonds show observed assortativity coefficients **(left)** and reciprocity coefficients **(right)**. Boxplots represent null distributions generated from a permutation test of 1,000 random networks with the same numbers of nodes, edges, and Spanish speakers like in the observed networks. The boxes represent the interquartile range (IQR); the line through the middle is the median. Whiskers extend to 1.5 × IQR; outliers are plotted as dots.

Our analysis represents a complete network comprising 408 egos and alters (48.5% women). Alters were more often Spanish speakers than egos (*n* = 336; 82.35% of alters and *n* = 59, 68.60% of egos were Spanish speakers). While we did not observe a non-random dissociation between Spanish and non-Spanish speakers based on whether individuals visited each other (i.e., the baseline network), we found evidence for language-based homophilic tendencies (i.e., positive assortativity) in the helping network as well as for negative language-based assortativity in the money network (Figure 5). In all three networks, reciprocity was much higher than expected by chance (Figure 5).

When considering the similarity in households’ connections across the networks, only 23.7% of the helping network’s connections were in common with the baseline (visiting) network. The money network was even more dissimilar, with only 6.8% of the connections in common with the visiting network and 4.4% with the helping one (Supplementary Table 6). Taken together, these results indicate that individuals assorted with different people for different purposes, but assortment based on language in none of the networks.

### Does Language Affect a Household’s

Given that the household is the unit of production and consumption (Kramer, 2005), the effect of the adult household head’s (*n* = 172) linguistic repertoire on their network position was considered. In the main text we show the independent effect of the linguistic competences of each household head on the household’s social network position (see Supplementary Figures 6–8 for the results of the same models but the existence of a joint effect for whether any household head was a fluent Spanish speaker).

**FIGURE 6:**
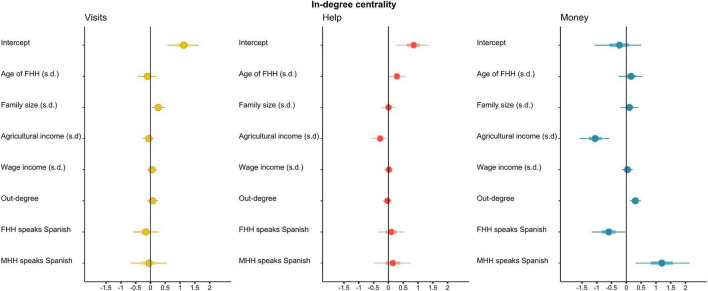
Posterior distributions of coefficients for the selected predictor variables for households’ in-degree centrality in the visiting network **(left)**, helping network **(middle)**, and money-lending network **(right)**. Points show median in-degree centrality, thick lines are 50% HPDIs, and thin lines are 90% HPDIs. A vertical line represents no effect. All continuous variables were standardized. The variables, top to bottom, refer to the number of hectares under cultivation available to the family, the size of the family, the age of the female household head (FHH) (SD), whether the male head of the household engaged in wage labor, out-degree, whether the female head of the household was a Spanish speaker and whether the male head of the household was a Spanish speaker. Note that as the visit network was drawn by women reporting their “out-degree,” in this network “in-degree” reflects the visits received by a household *only* from the female heads of other households. On the contrary, in-degree across the other two networks reflects help and money that households could obtain from any (member of any) other household.

Both in absolute terms and relative to the visiting network, households with Spanish-speaking FHHs and MHHs could rely on help from a similar number of individuals as those whose FHH or MHH was monolingual in Mayan [Estimate = 0.10, 90% HPDI (–0.32, 0.55) and Estimate = 0.13, 90% HPDI (–0.48, 0.56); Figure 6]. However, while households whose MHH spoke Spanish could obtain money loans from *more* individuals [Estimate = 1.20, 90% HPDI (0.31, 2.18)], those with FHH who spoke Spanish could borrow money from fewer people [Estimate =–0.59, 90% HPDI (–1.17,–0.01)]. That is, having a Spanish-speaking MHH and FHH had opposite effects on households’ in-degree centrality in the money network. When running the same models without controlling for out-degree, none of the coefficients changed.

To further investigate the effect of language on a household’s ability to access social support networks, we asked whether language affected the extent to which FHH invested in ties across the three networks. The results reveal moderate support for the view that Spanish-speaking FHH were willing to lend a week’s salary to fewer individuals (Estimate = –0.43; 90% HPDI [–0.84, –0.03]) (Supplementary Figure 6). However, they were willing to help the same number of alters with domestic duties (Estimate = 0.08, 90% HPDI: [–0.51, 0.71]) and to visit an equal number of individuals (Estimate = –0.16, 90% HPDI: [–0.60, 0.28]).

Then, we measured the effect of speaking Spanish on a household’s indirect ability to access village social support networks. Because we wanted to account for differences in the overall number of connections per household, we controlled in- and out-degree for indirect measures (BC and EC). Having Spanish-speaking MHHs or FHHs did not affect a household’s betweenness or eigenvector centralities (Supplementary Figures 4, 5). We also re-ran the analyses without an in- or out-degree control and found that the parameter estimates were not different from each other.

### Is Adopting a Mixed Economic Strategy Socially and Economically Advantageous for Households?

To determine whether new market opportunities are socially and/or economically advantageous, we examined whether households’ net income or social network outcomes were affected by their economic strategies. We found that wage income did not have an effect on the number of people from whom households’ received visits [Figure 6; Estimate = 0.09; 90% HPDI (–0.16, 0.34)], nor on whom they could rely for help [Estimate = 0.03; 90% HPDI (–0.22, 0.08)] or money [Estimate = 0.06; 90% HPDI (–0.23, 0.36)]. Nonetheless, it did have a positive effect on a household’s likelihood to act as a bridge between otherwise unconnected families (that is, its betweenness centrality) [Estimate = 0.42; 90% HPDI (0.14, 0.70); Supplementary Figure 4] as well as a marginal effect on the extent to which households were influential in the money network (their EC) [Estimate = 0.54; 90% HPDI (–0.10, 1.14); Supplementary Figure 6].

On the other hand, households with a greater agricultural income showed a reduction of in-degree centrality in both the helping [Estimate =–0.45; 90% HPDI (–0.87,–0.06); Figure 6] and money networks [Estimate =–1.70; 90% HPDI (–2.53,–0.9)].

To check the finding’s robusticity, given that whether the a MHH engaged in wage labor and spoke Spanish were significantly and positively correlated (Pearsons’ *r* = 0.36), we re-ran the same analyses excluding MHH wage labor status as a predictor. No change was observed on the effect of men’ or women’ linguistic repertoire on any of the response variables (degree, betweenness, or EC) in any of the three networks.

When evaluating the effects of a mixed economic strategy on household net income in 2017, we found that although the number of hectares under cultivation did not predict net income [Estimate =–2.33, 90% HPDI: (–4.95, 0.31)], the presence of a MHH who engaged in wage labor increased household income significantly [Estimate = 4.22, 90% HPDI: (0.97, 7.43); Supplementary Figure 8]. In the models with the linguistic profile of the FHH and MHH, the presence of a Spanish-speaking MHH had a marginal positive effect on the households’ net income [Supplementary Figure 7; Estimate = 3.19, 90% HPDI: (–0.27, 6.59)], while the presence of a Spanish-speaking FHH did not [Estimate =–1.87, 90% HPDI: (–5.78, 2.05)].

## Discussion

Our analyses point to several findings. First, in the Yucatec Maya setting, while the number of Spanish speakers has significantly increased over the course of market integration, this has not been at the expense of Yucatec Mayan; the latter remains universal in the village. This suggests that an increase in Spanish-speaking, rather than representing a the erosion of the indigenous language, might better be regarded as a strategy for households to pursue a mixed economy. In mixed economies, money earned in market activities is often redirected to support the maintenance of a traditional way of life and does not necessarily lead to the obsolescence of prior norms and values, including language (Poppel, 2010; BurnSilver et al., 2016; Ready, 2019).

Second, we find strong support for a relationship between speaking Spanish and the household participation in new economic opportunities that emerge as a result of market integration. In 2017, following the introduction of a road and economic development, households whose MHH engaged in wage labor gained clear economic benefits (Supplementary Figure 7). In this ethnographic context, although household agricultural wealth may not predict the probability of Spanish speaking, people living in households with no land under cultivation and committed to wage labor were more likely to be Spanish speakers. In turn, speaking Spanish was the strongest predictor of whether an individual would engage in wage labor, which for men was also a predictor of household net income (Figure 5 and Supplementary Figure 8). The association between economic strategies and an individual’s linguistic repertoire is also illustrated at the second time point, where younger individuals from large families were most likely to be both Spanish speakers and wage laborers. Living in a large family means that other siblings are available for agricultural work. Consequently, despite the fact that the younger generations are more likely to speak Spanish (mainly because they spend more time in school exposed to Spanish), being born into a large family further increases the relative benefit of learning Spanish to engage in wage labor.

Third, contrary to the conclusions based on general modeling approaches or regional-level analyses (Graves, 1967; Menéndez, 1981; Güemez Pineda, 1994; Cifuentes and Moctezuma, 2009; Fernando et al., 2010; Pfeiler et al., 2014; de León, 2019), our quantitative, detailed integration of individual- and household-level analyses illustrate that the role of education in promoting Spanish speaking has not led to a wide spread decrease in the use of Mayan. In other incipient bilingual contexts, education in the majority language is the result of low bargaining power in student–teacher coordination interactions and occurs in majority culture towns (as in cases of indigenous students attending majority culture boarding schools) (Bunce and McElreath, 2017). In Yucatán now, in remote rural communities, most children can complete primary and secondary education in their natal communities. Consequently, despite the use of Spanish in schools, they are educated exclusively with other Maya children. The Yucatec Maya in this rural area, while not isolated, live in a dispersed and an underpopulated region that is ethnically, socially, and economically homogeneous. Although they encounter non-Maya and Maya Spanish speakers in more urban contexts, for many villagers this is an occasional occurrence. This schooling situation, where indigenous students are educated in the national language in their natal communities, has also been documented among Matsigenka and Shipibo speakers in Peru, and found to in turn facilitate an “additive” pathway to bilingualism as opposed to a subtractive one (Tacelosky, 2001; Bunce, 2020).

Fourth, we find evidence for language-based homophily. Contrary to the predictions from the theories that propose phenotypic assortment based on language as being part of a universally evolved “ethnic psychology” (Chudek and Henrich, 2011; Henrich, 2011; Richerson et al., 2016), our results to not support a preferential association between same-language speakers. A recent study in a Yucatec Mayan–Spanish bilingual community found that speakers used linguistic competences to make social inferences about strangers (in particular, about their ethnic group membership) which was language-specific and dependent on their own language competences (Padilla-Iglesias et al., 2020). The strategic way in which individuals use language to guide their inferences and direct their behavior toward others, highlights the plasticity plastic nature of linguistic identity, that such plasticity may allow us to respond adaptively to the social world in which we live.

This is illustrated by our finding that, although individuals with similar linguistic repertoires were not more likely to visit one another, they were more likely to offer help to one another. This result is in line with suggestions that, while individuals may use language cues to seek in-group members this tendency should be more intense in helping compared to visiting interactions, as coordination failures and defections are likely more costly (Roberts, 2010; Moya and Boyd, 2015).

On the contrary, we observed a the *negative* assortment on language in the money network implies that Spanish speakers were more likely to lend money to Mayan monolinguals compared to other Spanish speakers. Among Tsimane, an indigenous population in Bolivia that has experienced an increasing market integration over the past half century, Gurven et al. (2015) found that more market-integrated individuals and households were more likely to redistribute their wealth with others from the village. The authors argued that this is possible because working for wages removes adult men from their home villages for periods of time, and was associated with higher variability in food production. This leaves households even more reliant on local support networks to buffer against the risk of resource shortfalls. Because the cost of giving is cheaper among wealthiest households (and in the same way as in Yucatán, more market-integrated Tsimane households were also wealthier), those households may redistribute their cash income as a means of gaining access to village helping networks. At the same time, Mayan monolinguals, who do not participate in wage labor, are reliant on others to access non-local resources and cash obtained from market activities.

Likewise, in Inuit populations, households engaged in a mixed economy had higher per capita incomes, lower rates of food insecurity and a greater median number of outgoing food sharing ties than those specialized in either a subsistence-based or a cash-based economy (BurnSilver et al., 2016; Ready and Power, 2018; Ready, 2019). Here too, those Maya households participating in wage work did not decrease their investment in local social networks, and indeed households with Spanish-speaking MHHs were willing to lend money to a greater number of villagers (Supplementary Figure 6).

Lastly, our study finds that bilingualism is notably gendered, with more men being bilingual relative to women. In 2017, in line with previous claims by Mijangos-Noh and Cardos-Dzul (2011), this perhaps can be attributed to access to education, and boys spending disproportionally more years in school. In 1992, the effect of gender was most likely due to male engagement in wage labor. Following hurricane Isidore in 2002 and local crop devastation, women began to participate in wage labor for the first time. The proportion of female wage laborers has remained fairly stable. In 2017, 5% of women over the age of 12 had wage labor jobs and were only unmarried women. However, this gendered pattern of bilingualism may not only be driven by sex differences in schooling and interaction with out-groups, but also by differential payoffs to do so; *specifically* in the network that has emerged as a consequence of market integration (i.e., the money network). While having a MHH who was fluent in Spanish positively affected both a household’s social (through their in-degree centrality) and economic (through their net income) outcomes in the money network, having a Spanish-speaking FHH was not associated with economic advantages.

This latter finding is consistent with many ethnographic accounts showing that men who are able to move between communities (in this case through wage labor and interacting with markets) are often accorded status in their home villages (Sahlins, 1972; Fitzhugh et al., 2011). For example, Coast Salish men with a greater number of between community ties were accorded more status within their communities, at least partially because these relationships provided access to non-local resources (Elmendorf, 1971).

Sex differences in social outcomes may could simply reflect that FHHs who spoke Spanish spent less time in the village and/or were less available. However, given that all 86 FHHs interviewed only participated in domestic work (they were neither wage laborers nor students), there is no *a priori* reason why this should be the case. In addition, although bilingual FHHs were willing to lend the equivalent of a week’s salary, they were not less likely to offer help or visit others, indicating that their investment in traditional local social networks is not different from that of their monolingual neighbors.

While linguistic affiliation has an important role in maintaining group cohesion (Richerson et al., 2016; Henrich, 2017), this study provides unique empirical evidence that within a single community language use may have different social outcomes for different individuals. Given that Maya women spend more time than men in the village, they have a central role in the building and consolidating local social relationships. Women may take the lead in sustaining traditional social networks, and in particular the helping network, where a positive assortment on language *was* observed. Whilst, as discussed above, it may be that women actively select partners *based* on their linguistic repertoires—i.e., using language as a marker of shared cultural affiliation or group commitment. But there is also the possibility that such an assortment is the result of opportunity costs for women who *acquired* competences in Spanish prior to establishing their own households. As Spanish competences were mostly acquired through education and work outside the village, that could mean that monolingual women who remained in the village had more opportunities to forge strong friendships with one another, which may be particularly important for accessing more costly forms of social support, such as help in domestic duties.

In the Yucatec Mayan study community, the roles of women in providing social capital contrasts with the roles of men in providing material capital. An example of the role of women in establishing community ties is the daily exchange of equal-size food servings between associated households, where females exchange a small portion of their midday meal with one another (Kramer, 2005). Other examples of women taking the lead in their households’ access to local networks *via* redundant or reciprocal sharing practices are common in the anthropological literature: Hiwi women from Venezuela routinely exchange identical quantities of wild tubers with one another (Gurven et al., 2000); and Martu and Meriam women from the Western Desert in Australia do so with monitor lizards and sardines, respectively [reviewed in Bliege Bird et al. (2018)]. Such exchanges may be particularly useful for signaling commitment to specific partnerships that are drawn on during unanticipated shortfalls. This is often referred to as social capital, meaning the ability of individuals to access and use the resources that are embedded in social networks (Bourdieu, 1986; Kaplan, 1996; Borgerhoff-Mulder et al., 2009).

Together, our results suggest that there are payoffs to accessing wage labor and economic opportunities that require Spanish-speaking, while also maintaining local social support networks. Rather than being a private good, social capital can be conceptualized as an emergent property of networks that enhances the solidarity and reproduction of groups (Coleman, 1988; Putnam, 2001). Treating social capital as a collective good sheds light on the potential function of language to demonstrate an individual’s commitment not just to others but to group affiliation. Given local gender roles, perhaps women benefit more from the signaling group commitment through language than men. In high-fertility populations, reifying in-group affiliation may have direct benefits. A previous study on this community shows that access to social support, in particular allocare, is a key for female fertility and infant survival (Kramer, 2005, 2014). While the assessment of differential linguistic behavior (in addition to language competence) across each of the networks goes beyond the scope of the present study, it would help clarify the mechanisms by which changes in individual-level patterns in language use following socioeconomic changes translate to shifts in population-level linguistic environments (MacKeigan and Muth, 2006).

## Conclusion

**FIGURE 7:**
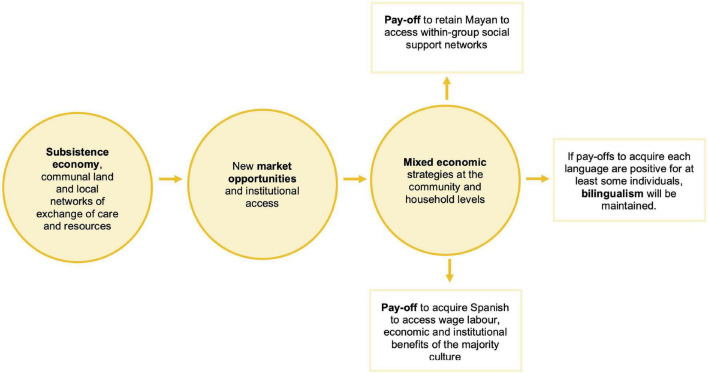
A schematic representation of the processes linking changes associated with market integration with economic and social influences on the payoffs to different linguistic repertoires. Payoffs predict those circumstances under which indigenous language use would be sustained and long-term linguistic diversity maintained, and those under which they are at risk.

In a 27-year period of socioeconomic changes in the Yucatán Peninsula, bilingualism in Yucatec Mayan and Spanish seems to be sustainable rather than transitionary. We have suggested that this is because each language allows individuals, households, and communities to obtain a complementary set of social and economic payoffs that are particularly advantageous in market integrating and mixed economies (Figure 7). Proficiency in the majority language is beneficial to facilitate household economic diversification, access wage work, and competitively participate in the market. At the community level, Spanish generates a novel dimension to local social capital by establishing a network of cash redistribution. Similarly, indigenous language competence signals an individual’s commitment to sharing and redistribution networks (childcare, help with domestic tasks, and lending and borrowing food and non-market goods), which generates adds to cohesion between the group members despite the individualization forces of market integration. Here, we emphasize a previously unexplored factor reinforcing the sustainability of minority languages, which are individual differences in the payoffs to becoming bilingual. Our results suggest that majority and indigenous language use, like many other cultural elements, are nuanced and depend on local payoffs. Moreover, a replacement trajectory may oversimplify the process of language acquisition.

## Data Availability Statement

The raw data supporting the conclusions of this article will be made available by the authors, without undue reservation.

## Ethics Statement

The studies involving human participants were reviewed and approved by University of Utah Ethics Committee and University of New Mexico Ethics Committee. The patients/participants provided their written informed consent to participate in this study.

## Author Contributions

KK collected the data. CP-I analyzed the data. Both authors designed the study, wrote the manuscript, and approved the final draft.

## Conflict of Interest

The authors declare that the research was conducted in the absence of any commercial or financial relationships that could be construed as a potential conflict of interest.

## Publisher’s Note

All claims expressed in this article are solely those of the authors and do not necessarily represent those of their affiliated organizations, or those of the publisher, the editors and the reviewers. Any product that may be evaluated in this article, or claim that may be made by its manufacturer, is not guaranteed or endorsed by the publisher.

## Footnotes

1 Note that unlike in the visiting and money networks where individuals report their “out-degree” (outgoing ties), in the helping network egos reported their “in-degree” (incoming ties).
