# Supplementary material for: The Role of Language in Structuring Social Networks Following Market Integration in a Yucatec Maya Population
Source: Front Psychol. 2021 Dec 16;12:656963. doi: 10.3389/fpsyg.2021.656963 (PMC8716436; doi:10.3389/fpsyg.2021.656963)
Supplement: Supplementary file 7 [file Data_Sheet_7.PDF]

<u>Supplementary Material for: The role of language in structuring social networks following market integration in a Yucatec Maya population</u>

By Cecilia Padilla-Iglesias and Karen L. Kramer

Table S1: Wage labor status of all individuals in the village census over 16 years of age in 2017.

	**Wage labor status**						
	Only agriculture	Agriculture and wage labor	Only wage labor	Student	Domestic work	Domestic work and wage labor	**Total**
Males	40.0% (n=79)	24.2% (n=48)	10.6% (n=21)	24.7% (n=49)	0.5% (n=1)	-	198
Females	1.0% (n=2)	0.5% (n=1)	2.6% (n=5)	16.1% (n=31)	77.1% (n=148)	2.6% (n=5)	192

Table S2: Descriptive statistics for Cohort 1 (1992) and Cohort 2 (2017) considered for the longitudinal analyses. Includes all individuals in the village aged 6 or above.

	**1992**		**2017**	
	No. children in the family	Hectares under cultivation	No. children in the family	Hectares under cultivation
Min.	0	0	0	0
Max.	11	5	15	40
Median	5	2.48	4	7.28
Mean	5.4	2.56	4.38	9.24
SE mean	0.15	0.06	0.14	0.36
95% CI mean	0.29	0.12	0.27	0.71
Variance	6.44	1.15	9.78	68.46
Std dev.	2.54	1.07	3.13	8.27

Table S3: Occupation of all individuals over 16 in 2017 according to their linguistic competencies.

	**Occupation**						
	Only agriculture	Agriculture and wage labour	Only wage labour	Student	Domestic work	Domestic work and wage labour	**Total**
Mayan monolinguals	28.3% (n=17)	-	-	1% (n=1)	66.7% (n=40)	3% (n=2)	60
Bilinguals	20.1% (n=61)	14.2% (n=43)	7.6% (n=23)	25.7% (n=78)	31.7% (n=96)	1% (n=2)	303

Table S4: Comparison of models assessing predictors of being fluent in Spanish in 1992 (left) and 2017 (right). Models with ΔWAIC <2 are highlighted in bold. Except for the intercept-only models, all others include the control predictors in addition to those reported in the table.

**1992**				**2017**			
Model	WAIC	weight	SE	Model	WAIC	weight	SE
**Control + HH size**	201.8	0.42	15.76	**Control + HH size x Age + Work of MHH**	236.9	0.35	24.77
**Control + HH size + Work of MHH**	202.7	0.28	16.01	**Control + Work of MHH**	237.4	0.28	24.01
Control model (Age + Sex + Years in education + HA under cultivation)	204.4	0.12	15.72	**Control model (Age + Sex + Years in education + HA under cultivation)**	237.9	0.21	23.76
Control + HH size x Age + Work of MHH	204.6	0.11	16.38	Control + HH size + Work of MHH	239.6	0.09	24.24
Control + Work of MHH	205.3	0.07	15.99	Control + HH size	240.2	0.07	24.12
Intercept - only	277.9	0	9.15	Intercept-only	521.2	0	15.49

Table S5: Comparison of models assessing predictors of working for wages in 1992 (left) and 2017 (right). Models with ΔWAIC <2 are highlighted in bold. Except for the intercept-only models, all others include the control predictors in addition to those reported in the table.

**1992**				**2017**			
Model	WAIC	weight	SE	Model	WAIC	weight	SE
**Control model (Age + Sex + Education + Family size + Land + Marital status)**	**36.5**	0.49	13.72	**Control + Spanish + Family size x Age**	**252.7**	0.88	24.1
**Control + Spanish + Family size x Age**	**37.5**	0.30	14.41	Control + Spanish	257.5	0.08	24.41
Control + Spanish	42.0	0.18	15.01	Control model (Age + Sex + Education + Family size + Land + Marital status)	258.6	0.05	23.6
Intercept-only	42	0.03	11.22	Intercept-only	338.4	0	18.96

Table S6: Jaccard indices between each pair of networks.

	Visits	Help	Money
**Visits**	NA	NA	0.068
**Help**	0.237	NA	0.044
**Money**	NA	NA	NA

Table S7: Descriptive statistics concerning the female heads of household who drew the social networks (n=86).

	Hectares under cultivation	Children ever had	Age
**Min**	0	0	17.4
**Max**	40	12	82.6
**Range**	40	12	65.2
**Median**	6	4	43.4
**Mean**	7.295	4.346	43.168
**SE mean**	0.741	0.316	1.538
**95% CI mean**	1.475	0.629	3.060
**Var**	44.505	8.104	191.485
**Std. Dev**	6.671	2.847	13.838

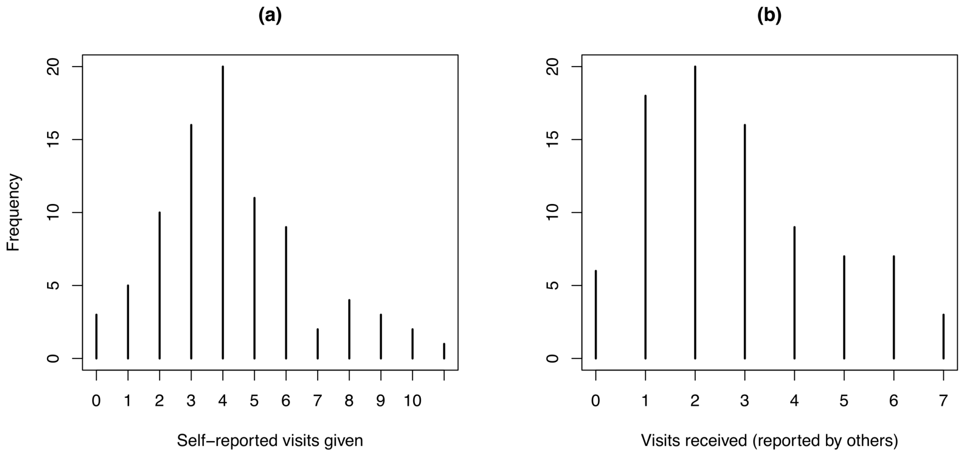

Fig. S1: Distributions of number of ties in the visiting network as reported by (a) participants themselves and (b) other participants.

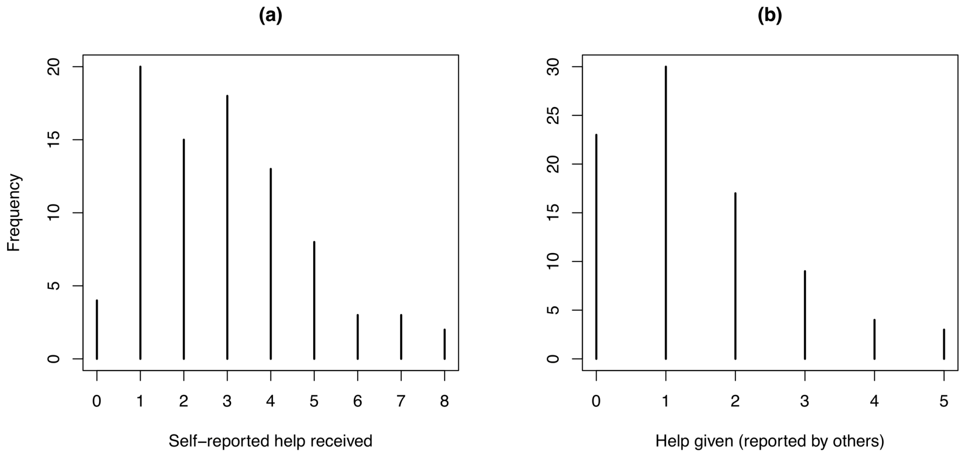

Fig. S2: Distributions of number of ties in the helping network as reported by (a) participants themselves and (b) other participants.

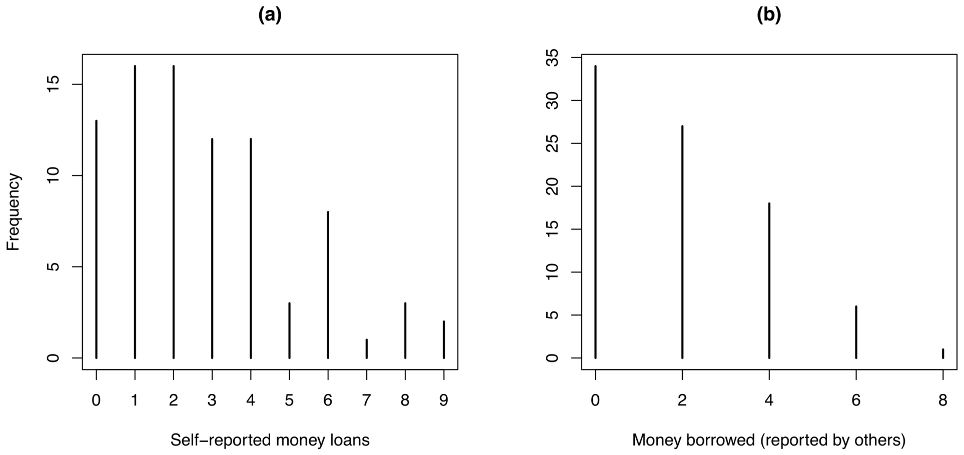

Fig. S3: Distributions of number of ties in the money network as reported by (a) participants themselves and (b) other participants.

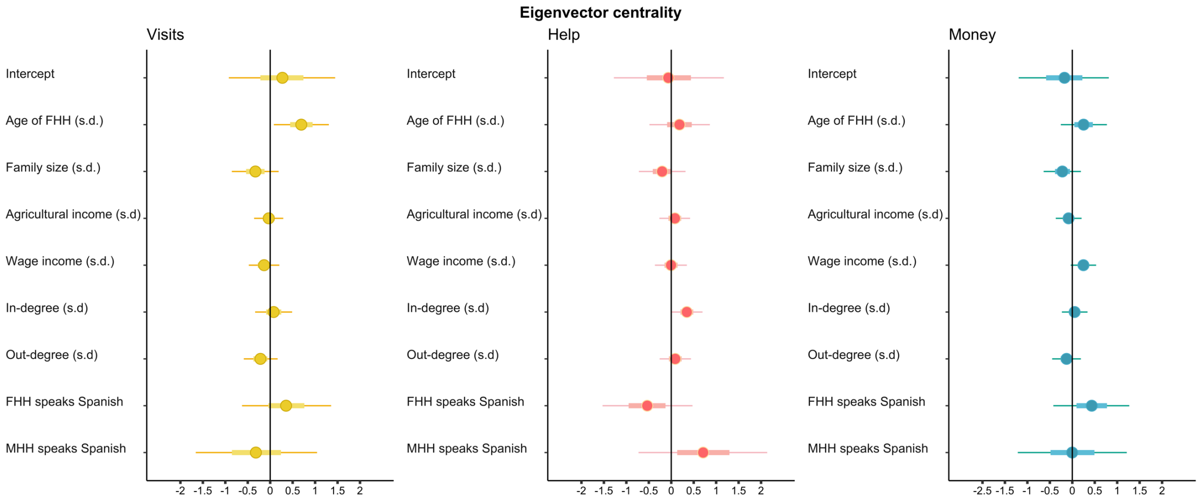

Fig S4: Posterior distributions of coefficients for selected predictor variables for households' eigenvector centrality in the visiting network (a), helping network (b) and money-lending network (c). 90% HPDIs are shown by the coloured lines. The numeric variables, including the response variable, were standardised. The dots represent the mean parameter estimates.

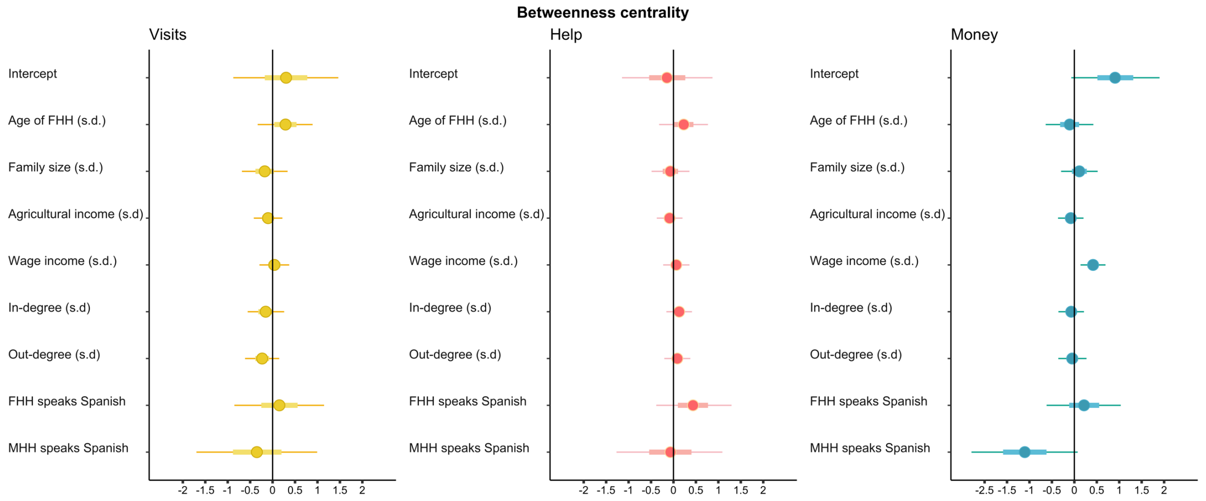

Fig S5: Posterior distributions of coefficients for selected predictor variables for households' betweenness centrality in the visiting network (a), helping network (b) and money-lending network (c). 90% HPDIs are shown by the coloured lines. The numeric variables, including the response variable, were standardised. The dots represent the mean parameter estimates.

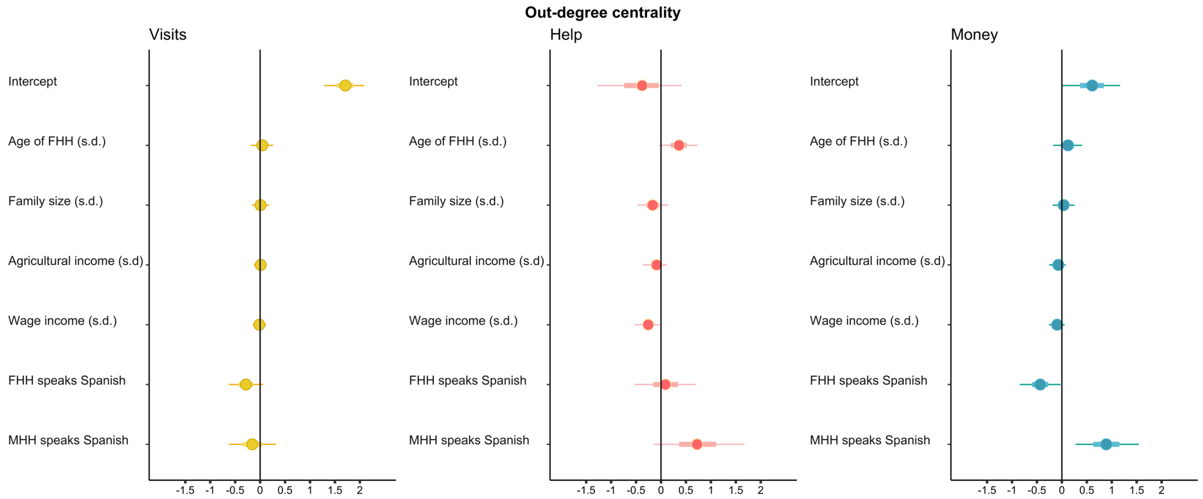

Fig S6: Posterior distributions of coefficients for selected predictor variables for households' out-degree centrality in the visiting network (a), helping network (b) and money-lending network (c). 90% HPDIs are shown by the coloured lines. The numeric predictor variables were standardised. Dots indicate mean parameter estimates.

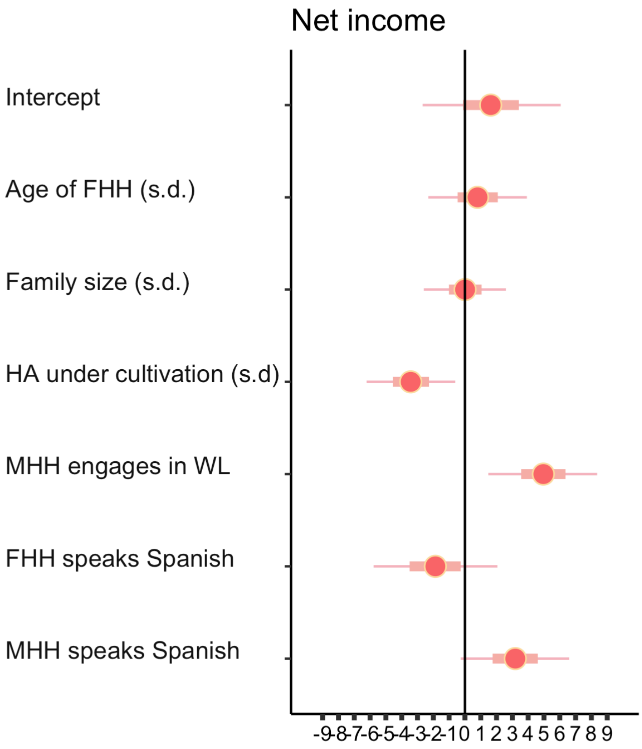

Fig S7: Posterior distributions of coefficients for selected predictor variables for households' net income. 90% HPDIs are shown by the coloured lines. The numeric predictor variables were standardised. Dots indicate mean parameter estimates.

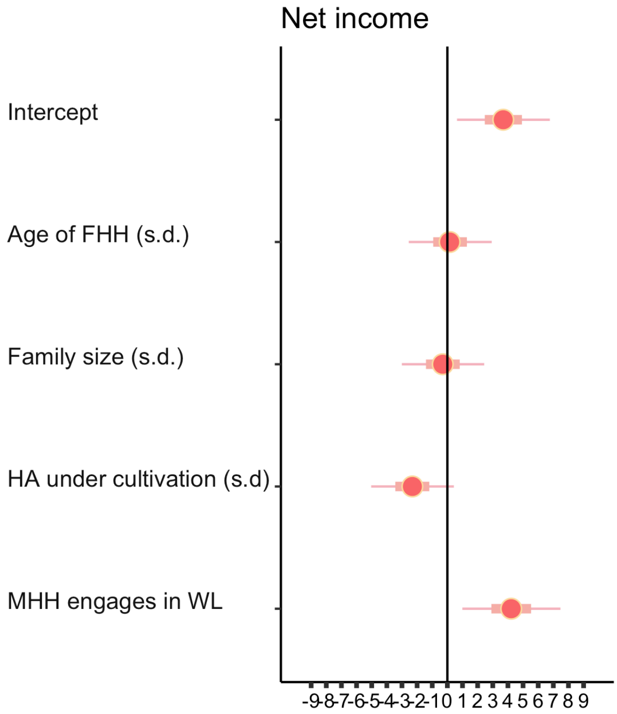

Fig S8: Posterior distributions of coefficients for selected predictor variables for households' net income. 90% HPDIs are shown by the coloured lines. The numeric predictor variables were standardised. Dots indicate mean parameter estimates.

Supplementary Text 1. Bayesian inference and Hamiltonian Monte Carlo

Bayesian inference is computationally intensive and therefore slow but permits greater modelling flexibility. However, the possibilities it allows of adding regularizing prior distributions to prevent extreme observations, of obtaining more informative results (a distribution of every possible parameter as opposed to a single value), and a precise quantification of uncertainty are often worth the increased computational cost (Gelman et al., 2013).

Utilizing Markov Chain Monte Carlo (MCMC) to obtain posterior distributions bypasses the need of assuming that the posterior distribution has a particular shape by sampling directly from it (McElreath, 2015, Chapter 9). Our choice of Hamiltonian MCMC stems from the fact that it performs much better than the most common MCMC algorithms, such as Gibbs sampling, which tend to fail in high dimensions (such as when including random intercepts), as they get stuck in local neighborhoods and poorly explore the posterior distribution (Neal, 2010; Betancourt, 2017).

Supplementary references

Betancourt, M. (2017). A conceptual introduction to Hamiltonian Monte Carlo. *ArXiv Preprint ArXiv:1701.02434*.

Gelman, A., Carlin, J. B., Stern, H. S., Dunson, D. B., Vehtari, A., & Rubin, D. B. (2013). *Bayesian data analysis*. Chapman and Hall/CRC.

McElreath, R. (2015). *Statistical rethinking: Texts in statistical science*. Boca Raton, FL: CRC Press.

Neal, R. M. (2010). *MCMC using Hamiltonian dynamics, Handbook of Markov Chain Monte Carlo (S. Brooks, A. Gelman, G. Jones, and X.-L. Meng, eds.)*. Chapman & Hall/CRC Press.
